# The composite redesign of humanity’s nature: a work in process

**DOI:** 10.1007/s11017-018-9440-5

**Published:** 2018-06-29

**Authors:** Lantz Fleming Miller

**Affiliations:** University of Twente, Enschede, Netherlands

**John Harris,** ***How to be good: The possibility of moral enhancement*, Oxford University Press, Oxford, 2016, 195** **pp, ISBN 978-0198707592**

**Yuval Noah Harari,** ***Homo Deus: A brief history of tomorrow*. Harvill Secker, London, 2016, 440** **pp, ISBN 978-1910701874**

**Willem H. Vanderburg,** ***Our battle for the human spirit: scientific knowing, technical doing, and daily living.*** **University of Toronto Press, Toronto, 2016, 440** **pp, ISBN 978-1487520359**

One of the most salient contemporary concerns in academic debates and pop culture alike is the extent to which new technologies may re-cast *Homo sapiens*. Species members may find themselves encased in a type of existence they deem to be wanting in comparison with their present form, even if the promised form was assured to be better. Plausibly, the concern is not merely fear of change or of the unknown, but rather it arises out of individuals’ general identification with what they are and what their friends and family are. In altering that identity beyond a point, they lose it and thus lose themselves in a kind of living death. The films about endemic zombieism seem to reflect such concerns about living death. Much of the recent literature on this issue of increasingly dissonant human/technology relations has largely come from medical ethics: from philosophers and physicians. Are we, as some contend, ethically obliged to incorporate these devices and methods into our being; or does such intervention violate some kind of tacit natural law? Already, as I have implied, the discussion reaches much further into the psychology of one’s identity, which has seen sparse examination in terms of this concern.

The three volumes reviewed in this essay offer widely differing perspectives on the relations between humans and new technology. The first by John Harris comes from an established tradition in medical ethics [1], providing another shaky rung in the endless climb up the ethics ladder. The second and third engage these issues from outside the conventional disciplinary bounds—Yuval Harari from the viewpoint of a historian [2] and Willem Vanderburg from that of an engineer [3]. Thus, the first volume serves as an effective foil—offering a common backdrop for evaluating the uncommonness of the latter two perspectives, which are not ethical in nature but social and cultural (through with ethical ramifications). The rare lessons to be taken from such social and cultural perspectives may help to illuminate the usual, more conventional medical-ethics approaches to pertinent problems relating to technology and human nature.

I start with Harris. A professional moralist, Harris has for many decades promoted the project most neutrally characterized as composite redesign of humanity’s nature (CROHN). This project is often narrowly deemed human enhancement (and some versions of it transhumanism); however, it is clearer to designate the project with less normatively loaded terminology. Harris has written so extensively promoting CROHN that this new volume’s bibliography lists only works that he himself has authored. I mention this fact because it plays into the book’s approach and method, as described below. In his work, Harris largely concentrates on CROHN projects—such as human cloning, genetic engineering, cognitive enhancement, and chimeras—and downplays the so-called ‘yuck’ factor in response to bioengineered beings, this factor being veritably immoral because it purportedly delimits the possibility for types of potential beings. While promoting a preponderance of CROHN techniques, he has also long debated other CROHN-favoring commentators regarding the use of projected pharmaceutical “moral bioenhancement” (MB). This new volume seems to be primarily focused on the ethics of various routes to moral improvement, as he finds the pharmaceutical method a threat to society and to the CROHN programs themselves.

I say this “seems” to be his focus because one of the central drawbacks to the volume is its sketchy, ununified structure. While only three of the twelve chapters are previously published essays, and while such reprinting is common and can support effective book-length arguments, justification for the volume’s lack of cohesion is not evident. Much of the book presents large passages from or summaries of the author’s earlier scholarship, and many of the footnoted citations are attributed to him. To read a book so pervaded by specters of the author’s previous work is, at best, an insignificant nuisance; at worst, it amounts to an elliptical experience for the reader. One would need to have at-hand access to the bibliography’s exclusive list of the author’s prior works, as well as unique insight into both what the author intends the reader to distill from each work and how to interpret this kernel within the context of the present volume. The result of this method, as reflected in the peculiar bibliography, reads like a patchy collage of the author’s oeuvre. As such, it is difficult to ascertain the precise theses of each chapter, much less that of the entire volume.

Despite this drawback in method and form, some content peeps through. As mentioned, the volume seems to unfold by differentiating MB from non-pharmacological moral enhancement (NONME, my acronym) and arguing against the former. In this respect, Chapters 4 and 5 are crucial—the latter critically describing MB and the former calling on the need for NONME, especially in the context of emerging technologies that may alter our species. The NONME that Harris holds is that “our chief hope of finding solutions to the most threatening sources of probable mass destruction and … our only proven forms of moral enhancement” are “science, innovation, and knowledge production, particularly education” [1, p. 74]. That is, cognitive enhancement precedes and induces moral enhancement. Opponents, such as Savulescu and Persson, contend the opposite: MB as soon as possible, then deal with the emerging cognitive enhancers. Yet Harris sees a grave threat to liberty here: “MB so far from raising consciousness, may well dull it to the point where the individual is no longer choosing [actions] at all” [1, p. 79].

When the reader gets to this point in Chapter 5, one can sense an overall theme materializing—one that, in retrospect, helps to contextualize Chapters 2 and 3. Buried in the last section of Chapter 2, which is astonishingly saltatory among a miscellany of topics, is the notion that moral enhancement must involve more than moral emotions, states of mind, intentions, or brain states; rather, it must consist in a “realistic calculation of consequence” [1, p. 37]. Such leaping about is quintessential in this book. In brief, after a jumble of sections about human rights, quotes from Shakespeare, and detours reviling Leon Kass, Chapter 2 at last simply offers a consequentialist stance. The reader then retrospectively feels out Chapter 3’s hermetic point that humans emphasize their humanness more than is morally good, especially since we should not morally mistreat the entities, such as chimeras, that may be born of emerging technologies. Otherwise, we would be guilty of the same offenses that stain our immoral ancestors, who refused to grant rights to minorities and women—hence the chapter title’s imperative to take ‘the human out of human rights.’

Although clear progress toward an overall argument remains obscure, in these chapters the reader is still able to interpolate—given lack of road signs—some kind of coherent endeavor. However, come the final section of Chapter 5, this hope for structure is frustrated by an inscrutable detour. After criticizing MB as intractable, Harris abruptly tosses in five pages about how humanity would best make moral progress by getting rid of males, the species’ single greatest moral burden. Certainly, he advocates doing so without murder—just select out male zygotes or y-sperm at the conception stage. With females’ better oxytocin capacity, a female-only world would be much more moral than the one we have today. Yet, notably, the author does not reconcile this authoritarian, paternalistic proposal for society with his vehemently argued position for freedom of choice as essential for moral action. Furthermore, the proposal for increasing levels of the neuroactive substance oxytocin in the population as a way to enhance morality does not mesh well with his criticism of MB, which also calls for raising levels of neuroactive substances in the population to enhance morality. At this juncture, with such patent contradiction, the book loses so much credibility as to threaten the validity of the remaining chapters. (A more charitable reading might hold that this section is instead intended as a joke; but then the remaining question about why such a joke would be relevant here, especially with no cues to signpost its humor, does not salvage the case for coherence.)

Indeed, the chapters that follow maintain the book’s saltatory structure. The thought experiment of an omniscient God Machine beaming into everyone’s brain would only stifle liberty; the notion that normal, non-pharmacological ethics is for ‘bad guys’ because ‘good guys’ know how to act does not contradict the MB position that cognition is not necessary for ethical behavior. Harris distances his arguments from Patricia Churchland’s views about molecular morality [4], then deplores the idea of mindreading as an infringement on freedom while apologizing for the invasion of privacy already extant in the (Big Data) cloud. He ends on an ambiguous note—half-cautionary, half-resigned—regarding the question of whether intelligent automata should be considered persons. Again, he presents a passel of interesting issues, but fails to pull a coherent thread through them, offering little in the way of clear argumentation and tending toward polemic with strangely inexact positions.

Harris’s new volume stands somewhere in the regions of traditional bioethical concerns for CROHN and exemplifies the range of such debates, even among CROHN-project supporters. Another new work, by the historian Yuval Harari [2], recognizes the ethical issues raised by CROHN programs, especially the extreme ones, without taking up the usual ethical approaches to these issues. His previous work, *Sapiens*, offers a skillfully concise telling of our species from our forager ancestors to early cultivators and thence industrial societies [5]. His present work attempts to project this story into the species’ future. If the effort succeeds anywhere, it is in corroborating the common notion that the future is not a parallel of the past, nor even an anti-parallel, even if the future steadily becomes the past. For humans at least, past and future are not broken mirrors of one another but very different concepts. Thus, to tell “the history of the future” may seem too cute an idea, and the reader may fear that the book will fall flat.

Yet—and it takes reaching the end of the long (67-page) first chapter to realize—the volume does become serious and more inviting, erasing fears of its potential cuteness. The author articulates his most basic and far-reaching assumption: “The following chapters discuss how humanism—the worship of humankind—has conquered the world. Yet the rise of humanism also contains the seeds of its downfall” [2, p. 65–66].

The major issue, contrasted with the main antagonist (humanism) in this attempted tale, is emerging technologies, especially those that do not exist but that many an engineer, a businessperson, and a philosophical cohort believe beyond a doubt will exist: the usual suspects—super intelligence, mind uploading, thoroughly virtual worlds, humanlike automata, and nanotech correction of any and all malady. These may appear, in some glistening eyes, to be something new on the planet. But for this volume, they are merely manifestations of the same old multi-century social-political phenomenon: humanism.

Humanism in this book is a “religion” [2, p. 66], a creed lasting about 300 years, that is, since the onset of the new scientific age. In placing *H. sapiens* at the center of the universe (*pace* Copernicus) and promoting the species’ self-worship, humanism has led the Earth to the Anthropocene, wherein the species is the most powerful force shaping the globe and life upon it. Supposedly, nature is nearly conquered; the species has almost entirely alienated itself from other lifeforms in the process, and now it can allegedly have as complete control of itself and the technical environment as the Greek gods had over their world. Yet “while the attempt to upgrade humans into gods takes humanism to its logical conclusion, it simultaneously exposes humanism’s inherent flaws” [2 p. 66]. In fact, “attempts to fulfil humanism result in its downfall” [2, p. 67]. Not only are these flaws exposed in the process of human upgrading, but they also undermine the humanistic program itself, threatening to drag life’s manifold into its pit. The book seems to offer hope that such analysis of humanism and its highly deleterious effects can help us, as free agents, to somehow neutralize this acid-like destructiveness.

The book’s intention, then, is clear and inviting. The problem lies not in its succoring hope, but in the fact that there is no singular creed of humanism. Rather, there seem to be multiple humanisms. Harari avers that, by the advent of the Twentieth Century, humanism had undergone a schism into three groups: orthodox liberal, socialist, and evolutionary. Yet there is still a nagging complication in speaking of humanism as the result of a schism as opposed to a multiplicity of ideologies or doctrines that have developed over millennia. To treat humanism as one monolithic movement, although certainly not unique among commentators, may be too much of a stretch. A historian of Harari’s expertise must understand the term “humanism” did not enter occidental vocabulary until Niethammer coined it in 1808, invoking Cicero’s *humanitas*, in a drive to reform education so as to inculcate values of civic virtue. Certainly, movements like Niethammer’s and later humanisms had existed for millennia, both in the West and in the East. But the perplexity for historians is the diachronic diversity of such movements. Why should they all be subsumed under the term “humanism”?

These movements seem to share with one another little more than the idea that members of the species should treat others well. Some humanists, such as Niethammer and even Cicero, would still allow a place for the Divine in human civic life; others would emphasize moral values derived purely from human interests. Some humanisms underscore the importance of understanding humanity’s position within nature and among other life forms; others encourage the notion that it is human destiny is to conquer nature. In a contemporary example, some commentators may indeed maintain, much in line with Harari’s assessment, that human-based values of exploration, discovery, and improvement dictate the species’ evolution toward a new type of species, harnessed by the drive to overwhelm or enslave nature. By contrast, at least one countermovement (*Pièce et main d’oeuvre*), maintains that it is anti-human to try to climb out of what their opponents deem to be an unworthy, faulty body and soul and to remake these in their own image of perfection.

In brief, Harari essentially asserts that humanity can either follow the (ersatz?) humanistic CROHN plan—a route he does not endorse—or buck humanism, sending a warning about the trend toward CROHN. It is simply not clear that attempts to fulfill humanism (or more accurately, humanisms) are what have led the species to the brink of likely self-destruction via techniques designed to guarantee consumers “immortality, bliss and divinity” [2, p. 278]. Certainly, one school of historical scholarship encourages the identification of sociopolitical trends to explain technological developments. Can such practice work for a ‘history of the future’ such as this one? Harari’s book does attempt to, by tying the twenty-first century search for technique-enabled bliss to “the traditional ideals of liberal humanism” [2, p. 279]:

Since humanism has long sanctified the life, the emotions and the desires of human beings, it’s hardly surprising that a humanist civilisation will want to maximise human lifespans, human happiness and human power. [2, p. 279]

In this quote and the rest of the paragraph, the book subtly reverts from the schism-fraught humanism to the monolithic humanism. Thus, at least in terms of this volume’s presentation, it remains unconvincing that the CROHN movement and its successes so far—by duping billions of consumers into buying high-technology products and thereby indirectly funding the movement—arise simply from the faulty philosophy of humanism.

The link to humanism seems to be thus: while liberal humanism enabled the scientific revolution and the technological wonders inspired by it, liberal free-market policies produced hyper-activated consumerism. Further, biological research increasingly shows that humans, like any organisms, are just mechanisms wholly determined and without free agency—thus undermining the very liberal humanism that gave rise to this scientific inquiry in the first place. Chapter 9’s great decoupling of liberal-humanism individuality from the results of liberal-humanism’s sciences makes the former obsolete and renders a crisis: (1) humans lose their economic and military status, rendering them useless and worthless; (2) for a while at least, the species as a whole will retain some value, while the individual loses all value; (3) for a while at least, the very few elite individuals upon whom the mechanized system depends will retain some value, making their extermination unlikely.

This prospective decoupling, which mirrors and intensifies Hannah Arendt’s concerns about automation, is indeed unsettling. The thesis merits the book’s convincing dissection and constitutes a large part of its warning. However, by the book’s end (on the Silicon Valley religion of Dataism), it is not apparent that either the extensive routing through a scapegoated humanism or the casting of CROHN as a demented humanism is necessary in order to make this warning sufficiently loud. In fact, because the humanisms are many, faulting humanism may detract even from efforts to help fence-sitters climb down and join the effort to defuse CROHN.

What exactly should be done in the face of CROHN scenarios that, as Harari implies, are totalitarian and very possibly genocidal remains unclear. Harari, for all his excellent insights (the humanism red herring aside), leaves readers with only three questions to think about as they go about their day: (1) Are we just algorithms? (2) Is consciousness not as important as intelligence? And (3) do we want our lives to be dominated by mechanisms that know more about each of us than we know ourselves? These are great concerns but are not emphasized in the book’s structure. Part of the author’s strategy may be to make the full implementation of CROHN so vivid and threatening as to disturb readers as much as any possible set of instructions can do.

Harris’ book reveals yet another anguishing angle here: though it represents only one of the few available explicitly philosophical works in support of CROHN, it is nonetheless poorly organized, lacks clear argumentation, and remains fraught with logical and critical-analytic pitfalls. In exemplifying what little substantial philosophical work has come out supporting CROHN, the Harris volume shows just how steeped the CROHN movement is in irrationality and in scant critical analysis.

A third recent volume, Vanderburg’s *Our Battle for the Human Spirit* [3]—though not about CROHN per se—offers concrete steps toward counteracting the widespread consumerist addiction to ever-newer devices. Vanderburg is a lifelong engineer and contributes the vocation’s rare and much-welcomed viewpoint on technology ethics. Besides his technical education, Vanderburg studied under Jacques Ellul, whose imprint is evident on almost every page—yet, curiously, without Ellul’s signature sense of insurmountable doom. Vanderburg sees gloom, but he also foresees a way to circumvent the gloom. One among many setbacks he identifies is specialty or discipline-based approaches to education and problem-solving. Such approaches pigeonhole the engineer into a narrow and particularized problem-solving space, which cannot fit the result to wider human needs. His work as an engineer, as well as his experience in dealing with many engineers, has made all too evident how the discipline-based approach serves to stifle the human spirit.

Another main contributor to the current deadened fact-driven industrial society is desymbolization—whereby activities and products render no integrated symbolic meaning to our idiosyncratic human identities. Little does one relate to anything else one does or what the rest of the universe means in terms of its relation to these actions. This is the challenge of secularization: “If technique destroys our being a symbolic species, it will likely disappear with us” [3, p. 85]. As connectedness with life and culture comes via technique instead of via others, “something essential about life is being filtered out” [3, p. 141]. The sciences and technique are both discipline-based, and they are equally separated from our needs. Scientists should thereby practice “a relative, and not an absolute, objectivity” [3, p. 95]—that is, one that is relative to our needs and the needs of the environment.

A solution may come from the process of resymbolization and extension beyond the discipline-based approach. Vanderburg offers the examples of Brazil’s Semco and Sweden’s Volvo Uddevala plant, which instigated worker-driven product assembly, wherein workers assembled entire automobiles in small teams. Such practice may seem to compromise efficiency, but in the end it proved sufficiently meaningful for the workers that efficiency was not significantly compromised. Yet, to return, symbolization would require a deliberate overhaul on the part of citizens, as well as on the parts of businesses and political leaders. Education would be a primary venue for restructuring discipline-based approaches as more culturally integrative, in the interest of resymbolization and restoring meaning to work, and in the interest of returning to real-life needs and the needs of the community and biosphere.

The author admits to having made inroads towards such goals in his work with the Canadian educational administration. Although many of those inroads have since been eroded, he maintains hope that change for the betterment of the human spirit is at least feasible—if only leaders would concur and act.

Vanderburg’s work may hold the most potential value of the three books discussed in this essay. He suggests how we as individuals, as citizens, and as members of a species can optimally respond to the ongoing barrage of new techniques. Vanderburg does not explicitly take on CROHN projects, although he mentions some CROHN techniques in passing. Yet these projects implicitly fall within the scope of his concern about how techniques and designs in the current cultural context are hampering the human spirit.

Unfortunately, despite the urgency of Vanderburg’s message, this book (the final of a five-part series) has stylistic shortfalls that can impede the promulgation of its message. The book’s style is at the same time thick and exceedingly repetitious, as though it were written spontaneously with little editing. The paragraphs are long and rambling, and it is hard to follow how the author’s thoughts connect. Certain phrases, while key, are reiterated to the point of irritation, such as the—albeit emphatically important—phrasing of concern for “human life, society, and the biosphere” (e.g., [3, p. 3]).

Nonetheless, the book’s message is sufficiently urgent and original that it is well worth a read by people on the many different sides of the CROHN debate. Unfortunately, few authors, such as Harari, have written non-antitechnology criticisms of CROHN, which has an enormous worldwide support system. As the three works reviewed here together make clear, a whole, fully coherent, definitive work on CROHN still has yet to materialize.
